# How does academic self-concept contribute to English language achievement in Chinese EFL college classes?

**DOI:** 10.3389/fpsyg.2026.1736480

**Published:** 2026-02-04

**Authors:** Xiao Zhou, Sidhu Gurnam Kaur

**Affiliations:** 1 School of Foreign Studies, Guangdong University of Science and Technology, Dongguan, China; 2 School of Education, MILA University, Nilai, Seremban, Malaysia

**Keywords:** academic buoyancy, academic self-concept, EFL, English language achievement, personal best, PLS-SEM

## Abstract

**Introduction:**

This study investigates how academic self-concept contributes to English language achievement among Chinese EFL college students, focusing on the mediating role of academic buoyancy and the moderating role of personal best. Guided by positive psychology theory, we clarify how self-belief, resilience, and self-referenced goal striving jointly shape English learning outcomes.

**Methods:**

Data were collected from 364 undergraduates enrolled in a private university in southern China. Participants completed a structured questionnaire. Partial Least Squares Structural Equation Modeling was employed to examine the measurement and structural models, assess mediation and moderation effects, and evaluate model fit and predictive validity.

**Results:**

The measurement model showed satisfactory reliability and validity; the structural model tested direct, mediating, and moderating effects. Results indicated that academic self-concept positively predicted both academic buoyancy and English language achievement. Academic buoyancy also positively predicted English language achievement, yielding a significant partial mediation from academic self-concept to English language achievement via academic buoyancy. Moderation analyses revealed a non-significant personal best effect on the academic self-concept to personal best path, but a significant negative moderation on the academic buoyancy to English language achievement path, suggesting that the achievement benefits of buoyancy are weaker when students’ personal best orientation is higher.

**Discussion:**

The findings highlight the pivotal roles of academic self-concept and academic buoyancy in enhancing English language achievement while revealing boundary conditions linked to personal best. Pedagogically, strengthening students’ self-belief and everyday academic resilience remains crucial; however, instructors should calibrate self-referenced goal setting to avoid inadvertently attenuating the performance yield of buoyant coping.

## 1 Introduction

In recent decades, Positive Psychology has emerged as a transformative paradigm in educational research, emphasizing learners’ strengths, motivation, and well-being rather than their deficiencies (Seligman, 2018; Macintyre et al., 2019; Wang et al., 2021). This movement has gained significant traction in educational contexts, leading to increased attention to positive psychology constructs, such as enjoyment, resilience or buoyancy, self-beliefs, and growth-oriented goals, which play vital roles in learners’ engagement and performance in L2 contexts, including Chinese EFL classrooms (Mercer, 2017; Mercer and Gregersen, 2023; Alazemi, 2024; Granziera et al., 2024; Guo et al., 2024; Cheng et al., 2025; Gao, 2025; Xiao and Liu, 2025). Among these factors, academic self-concept (ASC), academic buoyancy (AB), and personal best (PB) have garnered considerable scholarly interest due to their central role in predicting learning persistence and achievement outcomes (Liu, 2025; Putwain et al., 2024; Steinberg et al., 2024).

Meanwhile, China hosts one of the largest English-learning populations globally, with an estimated 400 million English learners across educational levels (Pan, 2019). English proficiency is widely recognized as a crucial academic and professional competency for Chinese undergraduates, as it serves both as a graduation requirement and a key determinant of employability and global competitiveness (Adamson and Xia, 2011; Cui et al., 2018). However, despite years of English instruction, many students continue to struggle with low English language achievement, as reflected in national benchmarks such as the College English Test Band 4 (CET-4) (Li, 2021; Song, 2023). Consequently, understanding the drivers of English learning success has become an urgent research priority in China’s higher education sector (Sang, 2023). To address these challenges, recent research in second language acquisition has increasingly turned to positive psychology as a new theoretical perspective for understanding learners’ motivation, well-being and outcomes (Mercer and MacIntyre, 2014; Derakhshan, 2025). This perspective emphasizes that students’ emotional resilience and positive self-beliefs play a crucial role in sustaining language learning success. This perspective emphasizes that students’ emotional resilience and positive self-beliefs play a crucial role in sustaining language learning success. Drawing on this framework, three key positive psychological constructs which including academic self-concept, academic buoyancy, and personal best have gained increasing attention for their significant roles in promoting achievement in educational contexts (Brumariu et al., 2023; Khajavy and Aghaee, 2024; Khasawneh et al., 2024). Academic self-concept refers to learners’ beliefs and evaluations about their own academic competence, which serve as the foundation for confidence and motivation in language learning (Arens and Jansen, 2016). Building on this, academic buoyancy captures students’ capacity to effectively manage and overcome everyday academic challenges and setbacks, enabling them to maintain persistence and engagement in their studies (Jahedizadeh et al., 2019). In addition, personal best, also referred as personal best goals, represents learners’ self-referenced striving to improve upon their previous performance, providing an adaptive and motivating standard that encourages continual growth (Martin and Liem, 2010). Collectively, these constructs have been identified as strong predictors of students’ engagement and achievement across educational contexts, thereby establishing the theoretical foundation for the present study.

Academic self-concept has long been recognized as a strong determinant of academic performance. It reflects learners’ beliefs about their competence in specific domains, influencing motivation, persistence, and effort regulation (Russ et al., 2025). In L2 learning, a positive ASC has been linked to increased self-efficacy, language enjoyment, and achievement (Chen et al., 2022; Kang and Wu, 2022). Empirical studies have demonstrated that students who perceive themselves as capable language learners are more likely to engage actively in learning tasks and achieve higher proficiency levels (Kang and Wu, 2022; Wu and Kang, 2023). For Chinese EFL learners, ASC is particularly significant because the English learning environment is predominantly exam-oriented and teacher-led, requiring strong internal motivation to sustain long-term learning engagement (Wu and Kang, 2023).

While ASC influences achievement, its effect may not be entirely direct. A strong sense of ASC fosters greater academic buoyancy in students, equipping them with the resilience to overcome challenges such as disappointing exam results or difficulties with language (Martin and Marsh, 2008). AB enables learners to maintain motivation and engage in adaptive coping when facing everyday academic stressors, which in turn supports ongoing academic performance (Martin and Marsh, 2008). Students with a strong ASC are more likely to remain persistent when facing language learning difficulties, as they tend to believe in their own ability to achieve academic success (Ulfah et al., 2024). AB, in turn, enables learners to sustain motivation and employ adaptive coping strategies when confronted with everyday academic stressors, thereby facilitating continuous learning progress (Martin and Marsh, 2008; Weißenfels et al., 2022). Empirical evidence further suggests that students who demonstrate higher levels of buoyancy are better able to regulate emotions, maintain engagement, and achieve stronger performance in English learning contexts (Wu, 2024). Therefore, AB may function as a mediating mechanism linking ASC to English language achievement, as learners with a strong sense of self-competence are more likely to develop the resilience needed to manage academic challenges effectively, which ultimately contributes to improved language outcomes.

In addition to mediation, individual motivational factors such as personal best may moderate the strength of relationships between ASC, AB, and academic achievement. Such goal orientation promotes adaptive motivation, task persistence, and positive affect, which buffer against the negative effects of failure or stress (Burns et al., 2018). Studies have shown that students with strong PB orientations exhibit higher engagement, self-efficacy, and academic resilience (Liem et al., 2012; Collie et al., 2016; Burns et al., 2018). In EFL learning, PB goals can sustain learners’ intrinsic motivation, helping them maintain progress even when encountering linguistic or contextual challenges (Jahedizadeh et al., 2021). Therefore, PB goals may strengthen the positive links between ASC, AB, and English achievement by fostering a mindset of continual self-improvement.

Although ASC, AB, and PB have each been studied extensively, there is limited research integrating all three constructs into a unified model to explain English language achievement in the Chinese EFL context. Most prior studies have examined these constructs independently, focusing either on self-beliefs (e.g., ASC) or on resilience (e.g., AB) without addressing their interconnections or the potential moderating role of PB (Guo et al., 2024). Furthermore, empirical evidence linking these constructs to objective performance indicators such as CET-4 scores remain scarce. Addressing this gap is critical for advancing theory and practice in L2 education, as it provides insights into how psychological strengths jointly promote academic achievement.

To address the identified gaps, this study examines how academic self-concept contributes to English language achievement among Chinese EFL college students, with academic buoyancy as a mediator and personal best as a moderator. Specifically, it aims to answer the following research questions:

RQ1: Is academic self-concept related to Chinese EFL college students’ English language achievement?

RQ2: Is academic self-concept related to students’ academic buoyancy in English learning?

RQ3: Does academic buoyancy mediate the relationship between academic self-concept and English language achievement?

RQ4: Does personal best moderate the relationship between academic self-concept and academic buoyancy?

By addressing these questions, this research seeks to clarify the dynamic interplay between self-beliefs, resilience, and motivational striving within the Positive Psychology framework, thereby offering a more comprehensive understanding of English achievement determinants among Chinese EFL college students.

## 2 Literature review

### 2.1 Theoretical framework

Positive psychology, a relatively new branch of psychology, emphasizes the scientific study and cultivation of positive emotions, personal strengths, and human well-being (Seligman and Csikszentmihalyi, 2000). Rather than focusing on deficits or pathologies, it examines the qualities that enable individuals to thrive and achieve fulfillment across various life domains, including education (Lopez, 2008; Seligman, 2011; Oxford, 2016). From a strength-based perspective, positive psychology views human challenges as opportunities for growth, emphasizing the development of resilience, self-belief, and motivation as pathways to optimal functioning and well-being (Seligman and Csikszentmihalyi, 2000; Boniwell and Tunariu, 2019). Within the context of English as a Foreign Language, positive psychology highlights how learners’ internal strengths, such as positive self-beliefs, adaptive motivation, and coping mechanisms, shape their engagement and achievement (Mercer, 2021). Specifically, the three interrelated constructs of academic self-concept (ASC), academic buoyancy (AB), and personal best (PB) are central to understanding learners’ performance from the positive psychology perspective.

Among these internal strengths, academic self-concept represents the cognitive foundation through which learners interpret their academic abilities and learning experiences (Oladrostam et al., 2025). Recent studies have shown that ASC is a significant predictor of EFL achievement, fostering motivation, persistence, and confidence in language use (Rost and Feng, 2024; Wang, 2024). Learners with a strong ASC tend to exhibit greater engagement and mastery orientation, which, in turn, enhances their language achievement (Liu et al., 2023; Souzandehfar and Ahmed Abdel-Al Ibrahim, 2023). Within positive psychology, ASC operates as an internal strength that promotes adaptive learning behaviors and emotional well-being in the classroom (Wang, 2024).

Beyond shaping learners’ beliefs about their academic competence, academic self-concept may also facilitate their ability to cope with everyday academic challenges (AB). Empirical evidence demonstrate that AB mediates the relationship between ASC and ELA, enabling learners with strong self-concept to sustain motivation and engagement when confronted with academic stress (Guo et al., 2024; Zhai, 2025). This aligns with the core principles of positive psychology, as buoyancy reflects learners’ resilience and optimism in navigating academic adversity.

Beyond the cognitive foundation of ASC and emotional regulation mechanism of AB, personal best (PB) serve as a critical motivational driver that links these two constructs to language achievement. When learners adopt PB, they channel their motivation toward outperforming their previous selves rather than competing externally, fostering autonomy and self-regulation (Tuna, 2024). Students striving for PB are more likely to experience positive emotions and sustained effort, both of which bolster buoyancy and academic performance.

Furthermore, positive psychology views learners’ achievement not merely as an outcome but as a reflection of learners’ flourishing—an integration of self-belief, resilience, and purposeful striving (Zhai, 2025). In this regard, ASC provides the cognitive foundation, AB the emotional regulation mechanism, and PB the motivational drive that collectively facilitate higher ELA. This integrated perspective echoes the shift in applied linguistics toward positive language education, which prioritizes learners’ emotional well-being, self-efficacy, and goal orientation (Dewaele et al., 2019; Mercer, 2021). By foregrounding the dynamic interplay among ASC, AB, and PB, the present study establishes a theoretically grounded basis for the subsequent hypothesis development.

### 2.2 Hypothesis development

Building on the theoretical framework outlined above, the following section develops hypotheses to examine the specific relationships among academic self-concept, academic buoyancy, personal best, and English language achievement.

Academic self-concept is recognized as a key psychological factor that shapes learners’ engagement, motivation, and academic outcomes. The relationship between ASC and language achievement has been well-documented in the literature. For instance, Cemal (2023) found that ASC, when combined with effective learning strategies, plays a crucial role in determining academic success in English language learning. Similarly, Syafri et al. (2019) reported that learners with a higher academic self-concept tend to demonstrate better English language competence, as their confidence in learning enhances effort, persistence, and task engagement. These findings align with earlier studies by Nosratinia et al. (2014), which identified ASC as a strong predictor of language performance among EFL learners. Despite these findings, there remains limited empirical work on this relationship in the context of Chinese EFL learners. To address this gap, the current study proposes the following hypothesis:

*H1*: Academic self-concept is positively related to English language achievement among Chinese EFL college students.

Academic self-concept and academic buoyancy are interrelated constructs that play crucial roles in explaining students’ adaptive functioning and persistence in academic settings. Empirical research consistently supports the idea that students with stronger academic self-concept demonstrate greater academic buoyancy and, consequently, higher academic achievement. For example, Colmar et al. (2019) found a significant positive association between self-concept and buoyancy, indicating that learners who perceive themselves as capable are better able to cope with daily academic pressures. Similarly, Ulfah et al. (2024) confirmed that academic self-concept, together with esteem support, significantly predicts buoyancy among mathematics students, reinforcing the view that self-belief strengthens emotional regulation and adaptive responses. Recent longitudinal findings further suggest that this relationship may be reciprocal rather than strictly unidirectional. Martin and Marsh (2020) found that buoyancy mitigates later academic adversity, indirectly reinforcing students’ self-beliefs, while Bostwick et al. (2022) identified bidirectional effects between buoyancy and motivational constructs across time. These results align with Putwain and Wood (2023), who noted that buoyancy and engagement form a self-sustaining feedback system. These findings collectively indicate that while academic self-concept acts as an antecedent to buoyancy, the relationship is interactive and self-reinforcing – strong self-belief fosters buoyancy, which in turn stabilizes and strengthens self-concept. Therefore, the following hypothesis is proposed:

*H2*: Academic self-concept is positively related to academic buoyancy.

According to the theoretical framework of positive psychology, students’ ability to effectively navigate academic challenges (AB) may expand their cognitive and emotional resources, thereby facilitating enhanced language learning outcomes. Existing studies provided substantial support for this proposition. Martin et al. (2010) have demonstrated that academically buoyant students tend to achieve higher academic performance across various disciplines. Specifically in language learning contexts, Putwain et al. (2020) found that resilience factors significantly predicted English achievement among secondary school students. Furthermore, recent investigations in Asian EFL settings have revealed that academic buoyancy positively correlates with language proficiency measures (Fu, 2023; Yang, 2023). However, while the majority of studies suggest a positive association between academic buoyancy and academic performance, some studies have reported non-significant or weak relationships between the two constructs. For example, Collie et al. (2015) conducted a longitudinal study with 2,971 Australian high school students and found that although academic buoyancy was initially associated with academic outcomes, its direct effect on achievement became non-significant after controlling for other variables. This indicates that academic buoyancy may not independently predict academic performance but could instead exert its influence with other factors. Given the mixed findings, this study proposes:

*H3*: Academic buoyancy is positively related to English language achievement.

Academic buoyancy has been widely recognized as a key affective mechanism linking students’ cognitive self-beliefs to academic outcomes. Prior research indicates that learners with stronger academic self-concept are more capable of sustaining motivation and coping with learning challenges through buoyancy, which ultimately enhances achievement. Empirical evidence supports this mediating mechanism: Colmar et al. (2019) verified that ASC significantly predicts both buoyancy and performance, while Ulfah et al. (2024) confirmed that higher self-concept and esteem support increase buoyancy under academic stress. Extending these findings, Guo et al. (2024) found that buoyancy mediates the relationship between motivation and academic performance, illustrating how positive self-beliefs are converted into adaptive coping mechanisms that enhance achievement. Recent evidence further strengthens this argument. Maqsood et al. (2025) revealed that buoyancy partially mediates the relationship between academic self-concept and academic outcomes, reducing self-handicapping behaviors and supporting sustained engagement. In a related study, Weißenfels et al. (2022) confirmed that buoyancy exerts an indirect influence on academic achievement through self-efficacy, underscoring its role as a motivational catalyst. Moreover, in the EFL context, Zhai (2025) demonstrated that academic buoyancy significantly mediates the link between learner engagement and English reading achievement, confirming its crucial role in language learning. Therefore, it is hypothesized that:

*H4*: Academic buoyancy mediates the relationship between academic self-concept and English language achievement.

Students with strong academic self-concept are more likely to demonstrate higher levels of academic buoyancy, but the degree to which this belief translates into resilient academic behavior depends on the presence of personal best. PB emphasize self-referenced improvement which striving to outperform one’s previous accomplishments rather than competing with others (Martin, 2006). They provide a motivational framework that directs self-belief toward mastery and persistence. When students with high ASC adopt PB, they are more capable of transforming confidence into perseverance and adaptability in the face of everyday academic challenges. This process fosters positive emotions, enhances self-regulation, and strengthens their academic buoyancy. Alazemi et al. (2023) demonstrated that PB striving significantly predicts academic buoyancy and emotional regulation among EFL learners. Similarly, Azemi et al. (2021) found that PB goals interact with motivational constructs to increase engagement and resilience. Li (2022) also confirmed that buoyancy serves as a self-regulatory mechanism that links self-beliefs and motivation to learning persistence. Collectively, these results suggest that PB goals do not simply predict buoyancy directly but instead strengthen the pathway between self-belief and adaptive functioning. Thus, the following hypothesis is proposed:

*H5*: Personal best moderates the relationship between academic self-concept and academic buoyancy.

Students who can recover effectively from academic challenges are more likely to benefit from PB, which motivate them to translate academic buoyancy into sustained academic effort and performance. When buoyant learners pursue PB, they channel their adaptability and persistence into self-regulated learning and achievement growth. Empirical evidence supports this moderating mechanism. Empirical research supports this moderating effect. Alazemi et al. (2023) demonstrated that PB striving and academic buoyancy jointly predict EFL learners’ performance and emotional regulation, confirming the motivational power of PB goals in language learning contexts. Aydın and Michou (2020) also found that self-determined motivation and PB goals significantly predict academic buoyancy and achievement, emphasizing that PB-driven learners better sustain their progress. Moreover, Jahedizadeh et al. (2021) revealed that PB goals positively relate to buoyancy and academic achievement among university language students, highlighting the synergistic role of PB striving and buoyancy in promoting success. Without PB goals, buoyant learners may recover from setbacks but lack the focus to sustain long-term improvement. Therefore, PB goals act as a moderating variable, strengthening the positive relationship between academic buoyancy and English language achievement. Given the above findings, this study proposes:

*H6*: Personal best moderates the relationship between academic buoyancy and English language achievement.

**Figure 1:**
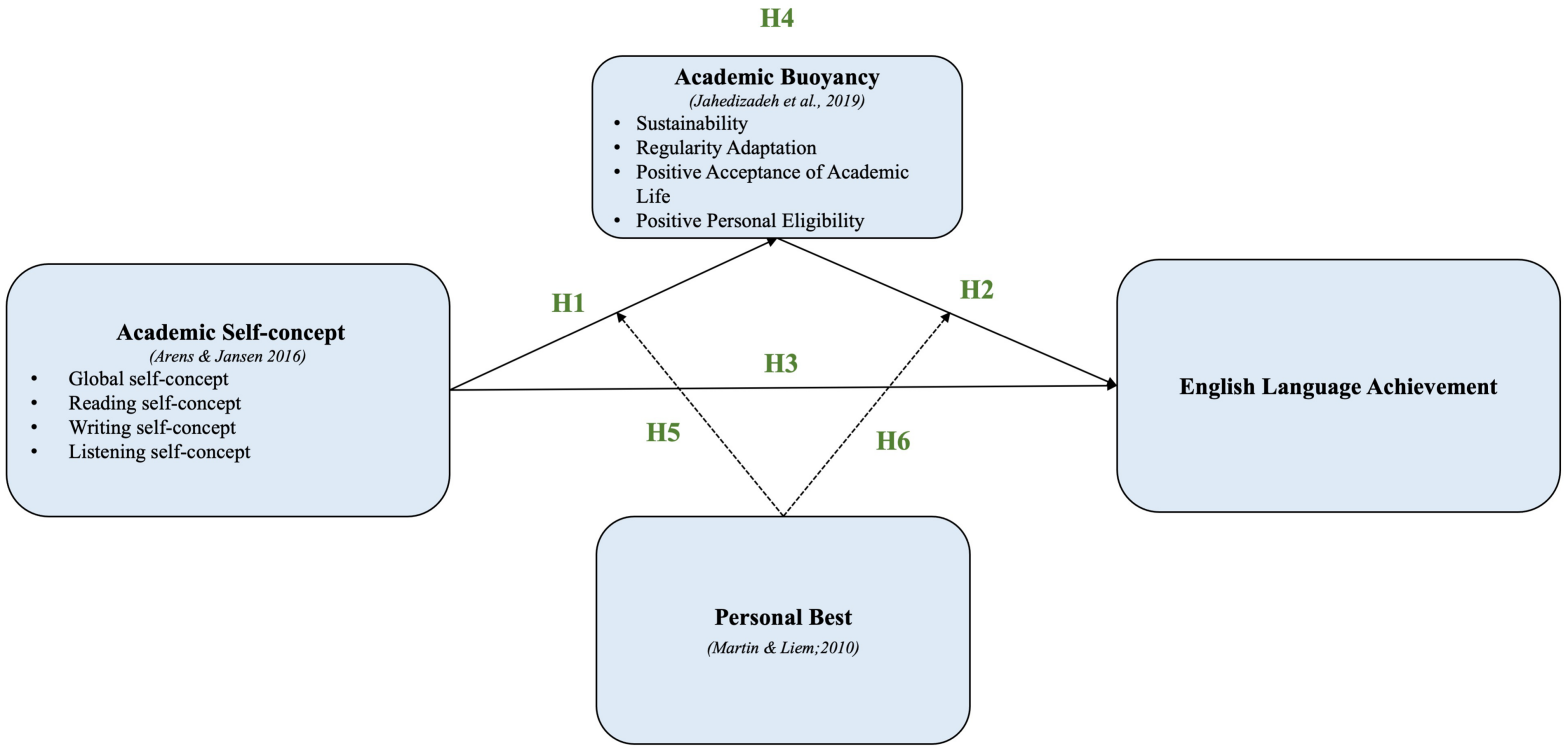
Conceptual framework.

Figure 1 illustrates the relationships within the posited conceptual model.

## 3 Methodology

### 3.1 Research design

This study employed a quantitative, cross-sectional survey research design to investigate the relationships among academic self-concept, academic buoyancy, personal best, and English language achievement among Chinese EFL college students. A correlational design was adopted, as the primary aim of the study was to test hypothesized associations, as well as mediation and moderation effects among latent variables. Given the complex structural relationships proposed in the conceptual model, partial least squares structural equation modeling (PLS-SEM) was selected as the primary analytical technique. PLS-SEM is particularly suitable for predictive research, complex models involving multiple latent constructs, and data that may not strictly meet multivariate normality assumptions (Hair et al., 2022).

### 3.2 Participants and sampling

The participants of this study were second-year undergraduate students enrolled at a private university in China. This population was selected based on both methodological relevance and practical feasibility. First-year students were excluded because most had not yet taken the College English Test Band 4 (CET-4), which served as the measure of English language achievement in this study. Third- and fourth-year students were also excluded, as many were engaged in internships or thesis writing, which could limit their availability and introduce additional variability unrelated to English learning.

A non-probability convenience sampling technique was employed to recruit participants. This approach was chosen due to its practicality in accessing a clearly defined population within a single institutional context and is commonly used in educational and psychological research where the primary aim is theory testing rather than population-level generalization (Etikan, 2016). Given the study’s focus on examining structural relationships among psychological constructs, convenience sampling was considered appropriate and methodologically justifiable.

Before statistical analysis, the dataset was thoroughly examined to detect and remove outliers, missing values, and straight-lining responses (Creswell and Creswell, 2022). Fifteen straight-lining cases and eight missing responses were excluded, resulting in a final sample of 364 valid questionnaires. This sample size exceeded the minimum of 200 recommended by Hair et al. (2022) for PLS-SEM, ensuring adequate statistical power for the analysis.

### 3.3 Demographic analysis

The demographic profile of the respondents is shown in Table 1, with 235 males (64.6%) and 129 females (35.4%). Most of them were 19 years old (55.5%), followed by 20 years old (27.2%). Regarding English study time, 59.9% of the students studied for less than 30 min per day, while 34.9% studied for 30 min to 1 hour. In terms of English proficiency, 60.4% identified as elementary, 37.1% as intermediate, and only 2.5% as advanced. Thus, the respondents were mainly male, aged between 19 and 20, and demonstrated relatively low English proficiency and limited self-study time, which is consistent with the characteristics of non-English-major students in private universities.

**Table 1:** Demographic characteristics of respondents.

Variables	Category	Frequency	Percentage
Gender	Male	235	64.6%
	Female	129	35.4%
Age	Under 18	2	0.5%
	18	32	8.8%
	19	202	55.5%
	20	99	27.2
	Over 20	29	8.0%
English study time	Under 0.5 h	218	59.9%
	0.5–1 h	127	34.9%
	1–2 h	14	3.8%
	2–3 h	1	0.3%
	Over 3 h	4	1.1%
English proficiency	Elementary	220	60.4%
	Intermediate	135	37.1%
	Advanced	9	2.5%

### 3.4 Instrumentations

Academic self-concept was adapted from the multidimensional scale developed by Arens and Jansen (2016), which conceptualizes academic self-concept as a hierarchical construct encompassing both general and skill-specific dimensions. In their model, four skill-specific self-concepts which including reading, writing, listening, and speaking along with a global self-concept dimension, were empirically validated. However, for the purpose of this study, only reading, writing, and listening self-concept scales, together with the global English self-concept, were retained to align with the CET-4 structure, which assesses reading, writing, and listening but not speaking. This alignment ensures conceptual coherence between the predictor (self-concept) and the criterion variable (English achievement). The adapted scale included 12 items, with three items representing each sub-dimension. All items were rated on a five-point Likert scale, ranging from 1 (strongly disagree) to 5 (strongly agree). An example statement is: “I am good at English.”

Academic buoyancy was measured using the academic buoyancy scale developed by Jahedizadeh et al. (2019). The scale comprises four dimensions: sustainability, regularity adaptation, positive personal eligibility, and positive acceptance of academic life. The original instrument contained 27 items, and respondents rated their agreement on a five-point Likert scale ranging from 1 (strongly disagree) to 5 (strongly agree). A sample item is: “I think I can cope with the pressure of studying English and my homework.”

Personal best was measured using the personal best scale developed and validated by Martin and Liem (2010). The scale consisted of four items, each rated on a five-point Likert-type response format (1 = strongly disagree to 5 = strongly agree). This instrument assesses students’ self-referenced striving for improvement and excellence in academic tasks. An example item includes: “When I work on my English language assignment, I try to do it better than I have done before.”

English language achievement was operationalized through the participants’ actual CET-4 scores, a standardized English proficiency examination administered nationwide by the Ministry of Education of China. The CET-4 is widely recognized as a valid indicator of college students’ English language proficiency and served as the dependent variable in this study. It provides an objective performance measure complementing the self-reported constructs.

To ensure validity and reliability, the questionnaire underwent expert review by a panel of three specialists. Their feedback was used to refine the item wording and structural alignment with the study’s theoretical framework. A pilot study with 102 participants was then conducted to examine the reliability and construct validity of the instrument through reliability testing and exploratory factor analysis. Based on the pilot results, eight problematic items from the AB scale were removed, while ASC and PB items remained unchanged. The final instrument comprised 35 items: 19 items for Academic Buoyancy (four sub-constructs), 12 items for Academic Self-Concept (four sub-constructs), and 4 items for personal best. Internal consistency reliability was assessed using Cronbach’s alpha, and all constructs demonstrated satisfactory reliability coefficients: PB (*α* = 0.924), ASC (*α* = 0.859), and AB (*α* = 0.793), exceeding the recommended threshold of 0.70 (Hair et al., 2022). These results indicate that the instrument was reliable, internally consistent, and suitable for further analysis.

### 3.5 Common method variance testing

To minimize potential common method variance (CMV) bias, both procedural and statistical remedies were applied in this study. Procedurally, the questionnaire was carefully designed to reduce respondents’ tendency to produce similar response patterns across items. Specifically, the constructs were placed on separate pages with intervals between them, as suggested by Podsakoff et al. (2003), to minimize the possibility of respondents answering consecutively with uniform response tendencies. Statistically, Harman’s single-factor test was conducted to assess the extent of CMV. All measurement items were loaded into an unrotated exploratory factor analysis, and the first factor accounted for 40.733% of the total variance, which is below the 50% threshold recommended by Podsakoff et al. (2003) and Hair et al. (2019). Therefore, CMV was not considered a serious issue in this study.

## 4 Data analysis and results

Prior to conducting the statistical analyses, the dataset was thoroughly screened to identify and remove outliers, missing values, and straight-lining responses (Creswell and Creswell, 2022). After comprehensive data cleaning and verification, 15 straight-lining cases and eight instances of missing values were detected and subsequently excluded from further analysis, resulting in a final sample size of 364 valid questionnaires.

Following data preparation, the analyses were performed systematically using SPSS 26.0 and SmartPLS 4.1.0.3, following established PLS-SEM procedures to ensure robust and reliable findings. Descriptive statistics were first computed in SPSS to summarize the demographic characteristics of the participants and the main study variables.

Next, Partial Least Squares Structural Equation Modeling (PLS-SEM) was employed as the primary data analysis technique due to its suitability for complex structural relationships and higher-order constructs (Hair et al., 2022). The proposed research model incorporated 2 second-order constructs: academic self-concept and academic buoyancy. Academic self-concept comprised four first-order constructs: (1) global self-concept (ASGS), (2) reading self-concept (ASRS), (3) writing self-concept (ASWS), and (4) listening self-concept (ASLS). Meanwhile, academic buoyancy consisted of four first-order constructs: (1) sustainability (ABS), (2) regularity adaptation (ABRA), (3) positive personal eligibility (ABPPE), and (4) positive acceptance of academic life (ABPAAL).

To represent these hierarchical relationships, a Hierarchical Component Model (HCM) was applied (Sarstedt et al., 2019; Hair et al., 2022). Both constructs were modeled as reflective–reflective Type I HCMs, where each higher-order construct was reflectively linked to its respective first-order constructs, and all lower-level indicators were measured reflectively (Hair et al., 2022). The HCMs were specified and evaluated using the two-stage approach. In the first stage, the measurement models of the first-order constructs were examined to verify indicator reliability, internal consistency, and convergent validity. Subsequently, latent variable scores were generated for the first-order constructs and used in the second stage to estimate the measurement models of the second-order constructs, thereby assessing the relationships between each higher-order construct and its dimensions.

After confirming the adequacy of the measurement models, the structural model was examined to test the hypothesized paths and interrelationships among the latent constructs.

### 4.1 Assessment of measurement model (first-order constructs)

In line with the recommendations of Hair et al. (2019), the measurement model was assessed using several reliability and validity indicators. At the construct level, Cronbach’s alpha (*α*), composite reliability (CR), and average variance extracted (AVE) were used as the primary indicators. Cronbach’s alpha and CR values exceeding 0.70 indicated satisfactory internal consistency, while AVE values above 0.50 confirmed adequate convergent validity. All first-order constructs demonstrated acceptable reliability and convergent validity, with Cronbach’s alpha values ranging from 0.79 to 0.92, CR values exceeding the recommended threshold of 0.70, and AVE values above 0.50 (see Table 2). These results indicate that the measurement model is reliable and suitable for subsequent structural model analysis.

**Table 2:** Assessment of measurement model of first-order constructs.

First-order constructs	Cronbach’s alpha	CR (rho_a)	CR (rho_c)	AVE
Positive acceptance of academic life	0.897	0.898	0.921	0.661
Positive personal eligibility	0.794	0.796	0.879	0.708
Regularity adaptation	0.79	0.797	0.877	0.704
Sustainability	0.916	0.916	0.933	0.665
Global self-concept	0.898	0.9	0.937	0.831
Listening self-concept	0.91	0.911	0.944	0.848
Reading self-concept	0.911	0.912	0.944	0.849
Writing self-concept	0.88	0.881	0.926	0.807
Personal best	0.871	0.871	0.912	0.721

To assess discriminant validity, the Heterotrait–Monotrait (HTMT) ratio was employed as the primary criterion. The HTMT approach has been widely recommended as a more rigorous and sensitive method for evaluating discriminant validity in variance-based structural equation modeling (Henseler et al., 2015). All HTMT values were below the conservative threshold of 0.85, indicating that the latent constructs were empirically distinct and that discriminant validity was adequately established. The HTMT results, presented in Table 3, provide sufficient evidence to support the discriminant validity of all constructs included in the measurement model.

**Table 3:** Heterotrait-monotrait (HTMT) ratio of first-order constructs.

First-order constructs	ABPAAL	ABPPE	ABRA	ABS	ASGS	ASLS	ASRS	ASWS
ABPAAL								
ABPPE	0.748							
ABRA	0.731	0.723						
ABS	0.776	0.695	0.69					
ASGS	0.506	0.481	0.51	0.509				
ASLS	0.448	0.389	0.483	0.403	0.768			
ASRS	0.379	0.33	0.358	0.375	0.822	0.715		
ASWS	0.414	0.372	0.444	0.451	0.78	0.779	0.709	
PB	0.682	0.736	0.576	0.602	0.424	0.342	0.299	0.345

### 4.2 Assessment of the measurement model (second-order constructs)

As shown in Table 4, the assessment of the second-order constructs demonstrated satisfactory measurement properties. All second -order construct composite reliability (CR) values were above 0.70 and average variance extracted (AVE) values exceeded 0.50 for both academic self-concept and academic buoyancy, providing evidence of satisfactory convergent validity. Discriminant validity of the second-order constructs was assessed using the Heterotrait–Monotrait (HTMT) ratio as the primary criterion. As presented in Table 5, all HTMT values were below the recommended threshold of 0.90, indicating that the second-order constructs were empirically distinct and that discriminant validity was adequately established.

**Table 4:** Assessment of measurement model of second-order constructs.

Second-order constructs	Cronbach’s alpha	Composite reliability (rho_a)	Composite reliability (rho_c)	AVE
AB	0.867	0.871	0.909	0.715
ASC	0.898	0.908	0.929	0.765

**Table 5:** HTMT ratio of second-order constructs.

Second-order constructs	AB	ASC
AB		
ASC	0.576	
PB	0.76	0.403

### 4.3 Structural model assessment

**Figure 2:**
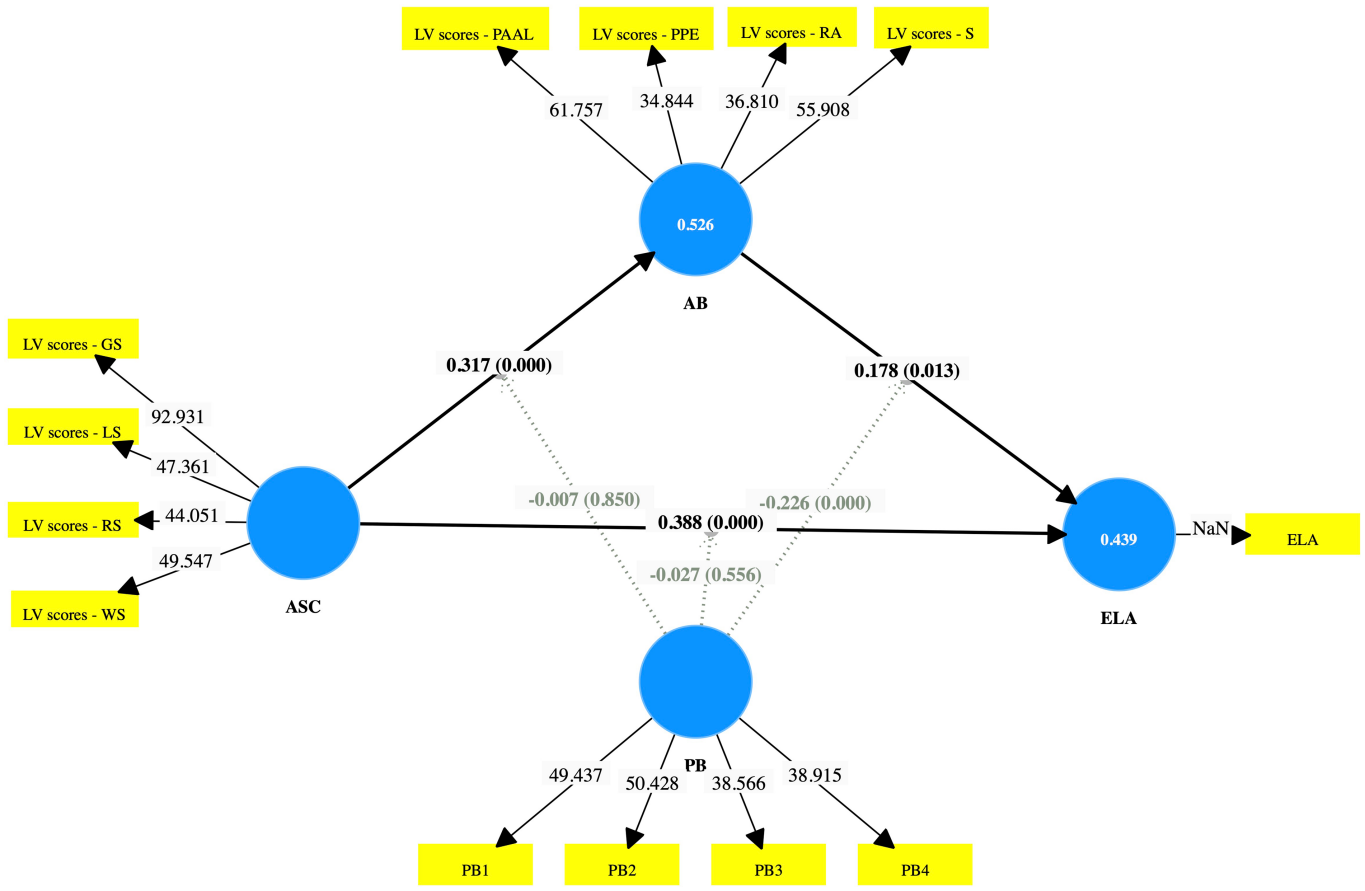
Structural model (path coefficients).

Following the establishment of the measurement model’s validity and reliability, the structural model was examined to evaluate the hypothesized relationships among constructs (see Figure 2). According to Hair et al. (2019), the strength and direction of relationships were assessed using path coefficients (*β*), with their significance tested through bootstrapping (5,000 subsamples). A path was considered significant if the t-value exceeded 1.96, the *p*-value was below 0.05, and the 95% confidence interval (CI) did not include zero. The coefficient of determination (*R*^2^) was used to measure the explained variance of the endogenous constructs. *R*^2^ values of 0.75, 0.50, and 0.25 indicate substantial, moderate, and weak explanatory power, respectively. The effect size (f^2^) was calculated to determine the impact of each exogenous construct on the endogenous constructs. Values of 0.02, 0.15, and 0.35 represent small, medium, and large effects, respectively. The predictive relevance (Q^2^), obtained using the blindfolding procedure, evaluates the model’s predictive accuracy. *Q*^2^ values of 0.02, 0.15, and 0.35 correspond to small, medium, and large predictive relevance (Hair et al., 2019). The Hypothesis testing results were displayed in Table 6.

**Table 6:** Hypothesis testing results.

Model pathway	*β*-values	Sample mean (M)	Standard deviation (STDEV)	T statistics (|O/STDEV|)	*p* values	95% CI [LL, UL]	Support
Direct effects							
H1: AB → ELA	0.178	0.183	0.072	2.489	0.013	[0.044, 0.324]	Yes
H2: ASC → AB	0.317	0.318	0.042	7.512	0	[0.234, 0.401]	Yes
H3: ASC → ELA	0.388	0.387	0.053	7.382	0	[0.282, 0.489]	Yes
Mediation effects							
H4: ASC → AB → ELA	0.057	0.059	0.025	2.252	0.024	[0.013, 0.112]	Yes
Moderation effects							
H5: PB × ASC → AB	−0.007	−0.006	0.038	0.189	0.85	[−0.081, 0.068]	No
H6: PB × AB → ELA	−0.226	−0.227	0.042	5.351	0	[−0.313, −0.146]	Yes

As shown in Table 6, the results revealed academic self-concept (ASC) showed a significant positive effect on English language achievement (ELA) (*β* = 0.388, *t* = 7.382, *p* < 0.001, CI not include zero), with a medium effect size (*f*^2^ = 0.181), thus supporting H1. Similarly, ASC had a significant positive effect on academic buoyancy (AB) (*β* = 0.317, *t* = 7.512, *p* < 0.001, CI not include zero), also with a medium effect size (*f*^2^ = 0.168), supporting H2. In addition, AB exerted a significant positive influence on ELA (*β* = 0.178, *t* = 2.489, *p* = 0.013, CI not include zero), although the effect size was small (*f*^2^ = 0.027), supporting H3.

The coefficient of determination (R^2^) and predictive relevance (Q^2^) of the endogenous constructs are shown in Table 7. The R^2^ value for academic buoyancy was 0.526, and the R^2^ for English language achievement was 0.439, suggesting moderate explanatory power for both constructs. Moreover, the Q^2^ values for AB (0.513) and ELA (0.362) suggest that the model exhibits large predictive relevance for both academic buoyancy and English language achievement, further supporting the model’s strong predictive capability.

**Table 7:** *R*^2^ and *Q*^2^ values for endogenous variables.

Second-order constructs	R-square	*Q*^2^ predict
AB	0.526	0.513
ELA	0.439	0.362

Mediation analysis was conducted following the procedure recommended by Hair et al. (2019), which involved examining the significance of both direct and indirect paths and calculating the Variance Accounted For (VAF) to determine the mediation type. First, the direct paths ASC → AB and AB → ELA were confirmed to be significant. Second, the indirect relationship between ASC and ELA via AB was tested, yielding a significant effect (*β* = 0.057, *t* = 2.252, *p* = 0.024, CI not include zero), thus supporting H4. Finally, the Variance Accounted For (VAF) was calculated to determine the strength of mediation. The indirect effect (0.057) relative to the total effect (0.444) produced a VAF of 12.80%, indicating partial mediation (see Table 8). This finding suggests that academic self-concept affects English language achievement both directly and indirectly through academic buoyancy, with about 13% of the total effect explained by the mediating pathway.

**Table 8:** Strength of mediation effects.

Mediation path	Indirect effect	Total effect	VAF	Decision
ASC → AB → ELA	0.057	0.444	12.80%	Partial mediation

Moderation analysis was further conducted to investigate the moderating role of PB on the relationships between ASC, AB, and ELA. The findings revealed that PB did not significantly moderate the relationship between ASC and AB (*β* = −0.007, *t* = 0.189, *p* = 0.850, CI include 0), thereby rejecting H5. However, PB significantly moderated the relationship between AB and ELA (*β* = −0.226, *t* = 5.351, *p* < 0.001, CI not include 0), supporting H6. The negative interaction effect indicates that higher levels of personal best weaken the positive effect of academic buoyancy on English language achievement. In other words, while academic buoyancy enhances English achievement, this positive influence becomes less pronounced among students who set extremely high personal standards, suggesting that excessive performance pressure may reduce the beneficial effect of buoyancy on learning outcomes.

## 5 Discussion

### 5.1 Academic self-concept as a foundational but context-sensitive predictor

The results revealed that ASC positively and significantly predicted both AB and ELA. This aligns with earlier research identifying ASC as a strong motivational and cognitive determinant of academic outcomes – one that drives students to persist, engage, and regulate their learning behaviors effectively, thereby supporting the reciprocal model of self-concept and achievement (Marsh et al., 2024; Sorjonen et al., 2024).

However, the magnitude of this relationship (*β* = 0.388) may be shaped by cultural and contextual factors. In Confucian-heritage societies like China, academic modesty and exam-centered pedagogy can temper students’ explicit self-beliefs (Lam, 2023), so its expression may differ from Western contexts dominated by self-enhancement norms (Heine and Hamamura, 2007). This suggests that interventions aiming to strengthen ASC in Chinese EFL classrooms must consider cultural expectations that discourage overt self-praise or individual assertion. Notably, ASC’s predictive power for ELA also reinforces the interdependence of affective and cognitive factors in language learning (Dewaele and Li, 2020). Yet, this study did not measure linguistic aptitude or anxiety directly, leaving open the question of whether ASC’s effects operate independently of these variables. Future longitudinal studies incorporating these constructs could clarify whether ASC exerts a causal influence or functions as a mediator between emotional and cognitive factors.

### 5.2 Academic buoyancy as a mediator: everyday resilience or adaptive persistence?

The mediating role of AB was statistically significant but modest in magnitude (*β* = 0.057, *p* = 0.024), indicating that buoyancy partially transmits the effect of ASC on ELA. Although the indirect effect is small, it is theoretically meaningful within the framework of positive psychology, where subtle psychological mechanisms can sustain motivation and engagement in everyday academic settings (Hair et al., 2022; Martin and Marsh, 2008). Rather than representing a strong causal driver, this modest mediation highlights the “everyday” nature of buoyancy – its role as a micro-level adaptive capacity that helps students recover from minor challenges such as feedback disappointment, test anxiety, or fluctuating confidence. This finding reinforces the conceptual distinction between resilience and buoyancy. While resilience concerns recovery from major setbacks, buoyancy reflects adaptive persistence during routine academic stress (Martin and Marsh, 2008). Nevertheless, the small effect size warrants a nuanced interpretation. It suggests that buoyancy alone may not dramatically transform achievement outcomes unless supported by other motivational and contextual factors such as teacher support or self-regulated learning (Kritikou and Giovazolias, 2022). Thus, educators should cultivate buoyancy alongside planning skills and emotional regulation to foster sustainable progress.

### 5.3 Personal best: motivational resource or hidden risk?

The moderation findings present a more complex picture. PB did not moderate the relationship between ASC and AB but negatively moderated the AB → ELA path. This finding contrasts with Martin and Liem’s (2010) assertion that PB goals universally enhance motivation and academic functioning. However, it is possible that when PB orientations become excessively strong, they may foster anxiety and perfectionistic tendencies, suggesting a critical threshold beyond which striving turns into self-imposed pressure with maladaptive consequences. This interpretation aligns with prior evidence that perfectionism is negatively associated with performance and tends to heighten anxiety (Lin, 2020; Liu et al., 2021), particularly in competitive academic contexts.

The non-significant moderation on the ASC to AB path also merits attention. It may suggest that self-belief already encompasses a sense of self-improvement, leaving limited variance for PB to explain. Alternatively, cultural moderation could play a role – self-effacing norms may prevent students from expressing strong PB goals even if they privately hold them. This raises theoretical questions about the universality of PB as a motivational construct: Is it equally adaptive in collectivist settings, or does it depend on how self-improvement is socially framed?

The negative moderation effect on relationship AB and ELA adds further nuance. It implies that as PB increases, the beneficial effect of buoyancy on achievement weakens. High-PB students may interpret minor academic fluctuations as personal failures, which undermines buoyant coping. This finding challenges the assumption that all forms of goal striving are beneficial. In fact, In fact, the broader literature on over-achievement and achievement goal theory warns that even ostensibly self-improvement goals may become maladaptive when they morph into excessive self-monitoring or a fear of under-performance (Lee et al., 2021; Diaconu-Gherasim et al., 2024). This study thus contributes critically by identifying a threshold effect: PB may support achievement up to a point but becomes counterproductive when internalized as self-pressure. Educators must therefore help students distinguish between healthy self-improvement and perfectionistic overstriving.

### 5.4 Limitations and future research directions

Although this study makes meaningful theoretical and empirical contributions to understanding how ASC contributes to English language achievement ELA, several limitations should be acknowledged, which also point to potential avenues for future research.

Firstly, the study employed a cross-sectional research design, which restricts the ability to infer causal relationships among ASC, AB, PB, and ELA. The relationships identified in this study represent statistical associations rather than directional causation. Future studies could employ longitudinal or experimental designs to trace how students’ self-concept and buoyancy evolve over time and to determine whether enhancing ASC or PB goals leads to sustainable improvements in English language achievement.

Secondly, the data were collected through self-reported questionnaires, which may be subject to social desirability bias or common method variance. Although statistical techniques such as Harman’s single-factor test and collinearity assessments were applied to minimize these risks, self-reported data may not fully capture students’ actual motivational states or behavioral engagement. Future research could incorporate mixed-methods approaches, including interviews, classroom observations, or digital learning analytics, to triangulate findings and enhance the ecological validity of the results.

Thirdly, the sample was limited to undergraduate students from a single private university in Guangdong Province, which may limit the generalizability of the findings to other regions or institutional contexts. Students in public universities or from different provinces may experience distinct academic pressures, learning resources, and English proficiency standards. To enhance external validity, future research should expand sampling to include multiple universities across China, or even conduct cross-cultural comparisons to explore whether the interplay among ASC, AB, and PB holds across different educational systems.

Fourthly, this study measured English language achievement primarily through CET-4 test scores, which, while standardized and reliable, may not capture the full range of communicative or intercultural competencies that constitute real-world English proficiency. Future studies could adopt multi-dimensional assessment methods, including speaking, writing, and online English learning performance metrics, to better represent the complexity of language achievement.

Finally, while this study focused on ASC, AB, and PB as psychological internal predictors, other contextual or affective variables, such as teacher support, peer interaction, or classroom climate, may also influence English learning success. Incorporating these external factors into an extended model could provide a more holistic understanding of how personal and environmental resources jointly shape students’ academic resilience and outcomes. Moreover, the moderating role of PB could be further examined through multi-group analysis, such as comparing high and low PB goal groups, to clarify how self-improvement orientations buffer against academic stress.

## 6 Conclusion

This study provides new insights into how academic self-concept, academic buoyancy, and personal best jointly shape English language achievement among Chinese EFL college students. The findings confirm that ASC is a fundamental psychological enabler of language achievement, exerting both direct and indirect influences through students’ buoyancy. Academic buoyancy emerged as a key mediating mechanism, translating self-belief into tangible academic outcomes. This highlights that students’ capacity to recover from setbacks is not only an adaptive emotional resource but also a motivational bridge connecting confidence to achievement. Interestingly, the moderating analysis revealed that PB did not strengthen the link between ASC and AB but negatively moderated the relationship between AB and ELA. This suggests that while striving for personal improvement remains beneficial, excessive self-comparison may dilute the positive influence of buoyancy on achievement. High PB orientation could shift students’ focus from adaptive recovery to self-imposed performance pressure, weakening the motivational advantage of buoyant coping. This research contributes to a deeper understanding of positive psychological mechanisms in EFL learning. It emphasizes that English language achievement is shaped not only by cognitive or instructional factors but also by learners’ beliefs, resilience, and goal orientations. Practically, the findings encourage educators to cultivate supportive classroom climates that enhance students’ self-concept, strengthen their academic buoyancy, and promote balanced personal-best striving. Future studies may extend these insights across broader cultural contexts and employ longitudinal approaches to capture how these motivational dynamics evolve over time.

## Data Availability

The raw data supporting the conclusions of this article will be made available by the authors, without undue reservation.

## References

[ref1] Adamson B. Xia B. (2011). A case study of the college English test and ethnic minority university students in China: negotiating the final hurdle. Multiling. Educ. 1:1. doi: 10.1186/2191-5059-1-1

[ref2] Alazemi A. F. T. (2024). Formative assessment in artificial integrated instruction: delving into the effects on reading comprehension progress, online academic enjoyment, personal best goals, and academic mindfulness. Lang Test Asia 14:44. doi: 10.1186/s40468-024-00319-8

[ref3] Alazemi A. F. T. Heydarnejad T. Ismail S. M. Gheisari A. (2023). A model of academic buoyancy, L2 grit, academic emotion regulation, and personal best: an evidence from EFL context. Heliyon 9:e13149. doi: 10.1016/j.heliyon.2023.e13149, 36785813 PMC9918774

[ref4] Arens A. K. Jansen M. (2016). Self-concepts in reading, writing, listening, and speaking: a multidimensional and hierarchical structure and its generalizability across native and foreign languages. J. Educ. Psychol. 108:646. doi: 10.1037/edu0000081

[ref5] Aydın G. Michou A. (2020). Self-determined motivation and academic buoyancy as predictors of achievement in normative settings. Br. J. Educ. Psychol. 90, 964–980. doi: 10.1111/bjep.12338, 31877237

[ref6] Azemi K. Shehni Yailagh M. Omidian M. (2021). The effect of personal best goals and social-emotional learning on academic engagement through the mediation of academic buoyancy. J. New Thoughts Educ. 17, 179–201. doi: 10.22051/jontoe.2021.31205.3036

[ref7] Boniwell I. Tunariu A. D. (2019). Positive psychology: theory, research and applications. Maidenhead: McGraw-Hill Education (UK).

[ref8] Bostwick K. C. Martin A. J. Collie R. J. Burns E. C. Hare N. Cox S. . (2022). Academic buoyancy in high school: a cross-lagged multilevel modeling approach exploring reciprocal effects with perceived school support, motivation, and engagement. J. Educ. Psychol. 114:1931. doi: 10.1037/edu0000753

[ref9] Brumariu L. E. Waslin S. M. Gastelle M. Kochendorfer L. B. Kerns K. A. (2023). Anxiety, academic achievement, and academic self-concept: meta-analytic syntheses of their relations across developmental periods. Dev. Psychopathol. 35, 1597–1613. doi: 10.1017/S0954579422000323 35491696

[ref10] Burns E. C. Martin A. J. Collie R. J. (2018). Adaptability, personal best (PB) goals setting, and gains in students’ academic outcomes: a longitudinal examination from a social cognitive perspective. Contemp. Educ. Psychol. 53, 57–72. doi: 10.1016/j.cedpsych.2018.02.001

[ref11] Cemal B. (2023). Predictive roles of language learning strategies, academic self-concept, gender and grade level in English language learning achievement. Psycho-Educ. Res. Rev. 12, 249–272. doi: 10.52963/PERR_Biruni_V12.N1.16

[ref12] Chen R. Iqbal J. Liu Y. Zhu M. Xie Y. (2022). Impact of self-concept, self-imagination, and self-efficacy on English language learning outcomes among blended learning students during COVID-19. Front. Psychol. 13:784444. doi: 10.3389/fpsyg.2022.784444, 35310252 PMC8931523

[ref13] Cheng J. Lu C. Xiao Q. (2025). Effects of gamification on EFL learning: a quasi-experimental study of reading proficiency and language enjoyment among Chinese undergraduates. Front. Psychol. 16:1448916. doi: 10.3389/fpsyg.2025.1448916, 40171076 PMC11958712

[ref14] Collie R. J. Martin A. J. Malmberg L.-E. Hall J. Ginns P. (2015). Academic buoyancy, student’s achievement, and the linking role of control: a cross-lagged analysis of high school students. Br. J. Educ. Psychol. 85, 113–130. doi: 10.1111/bjep.12066, 25604513

[ref15] Collie R. J. Martin A. J. Papworth B. Ginns P. (2016). Students’ interpersonal relationships, personal best (PB) goals, and academic engagement. Learn. Individ. Differ. 45, 65–76. doi: 10.1016/j.lindif.2015.12.002

[ref16] Colmar S. Liem G. A. D. Connor J. Martin A. J. (2019). Exploring the relationships between academic buoyancy, academic self-concept, and academic performance: a study of mathematics and reading among primary school students. Educ. Psychol. 39, 1068–1089. doi: 10.1080/01443410.2019.1617409

[ref17] Creswell J. W. Creswell J. D. (2022). Research design: qualitative, quantitative, and mixed methods approaches. Thousand Oaks, CA: SAGE Publications.

[ref18] Cui S. Pan K. Ye Y. (2018). Language ability or personality works?: the return to possessing a global English test certificate for college graduates in China. ECNU Rev. Educ. 1, 74–101. doi: 10.30926/ecnuroe2018010204

[ref19] Derakhshan A. (2025). Revisiting research on positive psychology in second and foreign language education: trends and directions. Lang. Relat. Res. 13, 1–43. doi: 10.52547/LRR.13.5.2

[ref20] Dewaele J.-M. Chen X. Padilla A. M. Lake J. (2019). The flowering of positive psychology in foreign language teaching and acquisition research. Front. Psychol. 10:2128. doi: 10.3389/fpsyg.2019.02128/full 31607981 PMC6769100

[ref21] Dewaele J.-M. Li C. (2020). Emotions in second language acquisition: a critical review and research agenda. Foreign Lang. World 196, 34–49.

[ref22] Diaconu-Gherasim L. R. Elliot A. J. Zancu A. S. Brumariu L. E. Măirean C. Opariuc-Dan C. . (2024). A meta-analysis of the relations between achievement goals and internalizing problems. Educ. Psychol. Rev. 36:109. doi: 10.1007/s10648-024-09943-5

[ref23] Etikan I. (2016). Comparison of convenience sampling and purposive sampling. Am. J. Theor. Appl. Stat. 5, 1–4. doi: 10.11648/j.ajtas.20160501.11

[ref25] Fu L. (2023). Social support in class and learning burnout among Chinese EFL learners in higher education: are academic buoyancy and class level important? Curr. Psychol., 1–15. doi: 10.1007/s12144-023-04778-9, 37359569 PMC10215065

[ref26] Gao F. (2025). Academic buoyancy and achievement among EFL learners: the mediating roles of hope and enjoyment. Acta Psychol. 260:105595. doi: 10.1016/j.actpsy.2025.105595, 40991972

[ref27] Granziera H. Collie R. J. Martin A. J. Caldecott-Davis K. (2024). Adaptability and buoyancy: investigating their unique associations with students’ wellbeing and academic achievement. Educ. Psychol. 44, 927–945. doi: 10.1080/01443410.2024.2418637

[ref28] Guo H. Zhou Z. Ma F. Chen X. (2024). Doctoral students’ academic performance: the mediating role of academic motivation, academic buoyancy, and academic self-efficacy. Heliyon 10:e32588. doi: 10.1016/j.heliyon.2024.e32588, 39021903 PMC11252879

[ref29] Hair J. F. Hult G. T. M. Ringle C. M. Sarstedt M. (2022). A primer on partial least squares structural equation modeling (PLS-SEM). (3rd ed.). Los Angeles, CA: Sage.

[ref30] Hair J. F. Risher J. J. Sarstedt M. Ringle C. M. (2019). When to use and how to report the results of PLS-SEM. Eur. Bus. Rev. 31, 2–24. doi: 10.1108/EBR-11-2018-0203

[ref31] Heine S. J. Hamamura T. (2007). In search of east asian self-enhancement. Personal. Soc. Psychol. Rev. 11, 4–27. doi: 10.1177/1088868306294587, 18453453

[ref32] Henseler J. Ringle C. M. Sarstedt M. (2015). A new criterion for assessing discriminant validity in variance-based structural equation modeling. J. Acad. Mark. Sci. 43, 115–135. doi: 10.1007/s11747-014-0403-8

[ref33] Jahedizadeh S. Ghonsooly B. Ghanizadeh A. (2019). Academic buoyancy in higher education: developing sustainability in language learning through encouraging buoyant EFL students. J. Appl. Res. High. Educ. 11, 162–177. doi: 10.1108/JARHE-04-2018-0067

[ref34] Jahedizadeh S. Ghonsooly B. Ghanizadeh A. (2021). A model of language students’ sustained flow, personal best, buoyancy, evaluation apprehension, and academic achievement. Porta Linguarum 35, 257–275. doi: 10.30827/portalin.vi35.15755

[ref35] Kang X. Wu Y. (2022). Academic enjoyment, behavioral engagement, self-concept, organizational strategy and achievement in EFL setting: a multiple mediation analysis. PLoS One 17:e0267405. doi: 10.1371/journal.pone.0267405, 35486654 PMC9053798

[ref36] Khajavy G. H. Aghaee E. (2024). The contribution of grit, emotions and personal bests to foreign language learning. J. Multiling. Multicult. Dev. 45, 2300–2314. doi: 10.1080/01434632.2022.2047192

[ref37] Khasawneh M. A. S. Ismail S. M. Hussen N. (2024). The blue sky of AI-assisted language assessment: autonomy, academic buoyancy, psychological well-being, and academic success are involved. Lang Test Asia 14:47. doi: 10.1186/s40468-024-00318-9

[ref38] Kritikou M. Giovazolias T. (2022). Emotion regulation, academic buoyancy, and academic adjustment of university students within a self-determination theory framework: a systematic review. Front. Psychol. 13:1057697. doi: 10.3389/fpsyg.2022.1057697, 36524164 PMC9746693

[ref39] Lam C.-M. (2023). A confucian approach to teaching humility. Educ. Philos. Theory 55, 207–216. doi: 10.1080/00131857.2022.2112032

[ref40] Lee M. Bong M. Kim S. (2021). Effects of achievement goals on self-control. Contemp. Educ. Psychol. 67:102000. doi: 10.1016/j.cedpsych.2021.102000

[ref41] Li J. (2021). Perceived effects of CET4 test preparation, language ability, and test performance: an exploratory study of Chinese EFL learners. Lang. Educ. Assess. 4, 38–58. doi: 10.29140/lea.v4n2.480

[ref42] Li W. (2022). Resilience among language learners: the roles of support, self-efficacy, and buoyancy. Front. Psychol. 13:854522. doi: 10.3389/fpsyg.2022.854522, 35360572 PMC8962401

[ref43] Liem G. A. D. Ginns P. Martin A. J. Stone B. Herrett M. (2012). Personal best goals and academic and social functioning: a longitudinal perspective. Learn. Instr. 22, 222–230. doi: 10.1016/j.learninstruc.2011.11.003

[ref44] Lin L. (2020). Perfectionism and writing performance of Chinese EFL college learners. Eng. Lang. Teach. 13:35. doi: 10.5539/elt.v13n8p35

[ref45] Liu R. (2025). Psychological resources for academic buoyancy: the roles of growth mindset and emotional intelligence in Chinese university students. Front. Psychol. 16:1580929. doi: 10.3389/fpsyg.2025.1580929, 40538481 PMC12176830

[ref46] Liu Q. Du X. Lu H. (2023). Teacher support and learning engagement of EFL learners: the mediating role of self-efficacy and achievement goal orientation. Curr. Psychol. 42, 2619–2635. doi: 10.1007/s12144-022-04043-5

[ref47] Liu C. He J. Ding C. Fan X. Hwang G.-J. Zhang Y. (2021). Self-oriented learning perfectionism and English learning burnout among EFL learners using mobile applications: the mediating roles of english learning anxiety and grit. Learn. Individ. Differ. 88:102011. doi: 10.1016/j.lindif.2021.102011

[ref48] Lopez S. J. (2008). Positive psychology: exploring the best in people. New York, NY: Bloomsbury Publishing.

[ref49] Macintyre P. D. Gregersen T. Mercer S. (2019). Setting an agenda for positive psychology in SLA: theory, practice, and research. Mod. Lang. J. 103, 262–274. doi: 10.1111/modl.12544

[ref50] Maqsood F. Yasin H. Farooq S. F. (2025). Academic self-handicapping, academic buoyancy, and self-concept in college students. Contemp. J. Soc. Sci. Rev. 3, 1630–1640. doi: 10.63878/cjssr.v3i3.1155

[ref51] Marsh H. W. Guo J. Pekrun R. Lüdtke O. Núñez-Regueiro F. (2024). Cracking chicken-egg conundrums: juxtaposing contemporaneous and lagged reciprocal effects models of academic self-concept and achievement’s directional ordering. Educ. Psychol. Rev. 36:53. doi: 10.1007/s10648-024-09887-w

[ref52] Martin A. J. (2006). Personal bests (PBs): a proposed multidimensional model and empirical analysis. Br. J. Educ. Psychol. 76, 803–825. doi: 10.1348/000709905X55389, 17094887

[ref53] Martin A. J. Colmar S. H. Davey L. A. Marsh H. W. (2010). Longitudinal modelling of academic buoyancy and motivation: do the 5Cs hold up over time? Br. J. Educ. Psychol. 80, 473–496. doi: 10.1348/000709910X486376, 20170601

[ref54] Martin A. J. Liem G. A. D. (2010). Academic personal bests (PBs), engagement, and achievement: a cross-lagged panel analysis. Learn. Individ. Differ. 20, 265–270. doi: 10.1016/j.lindif.2010.01.001

[ref55] Martin A. J. Marsh H. W. (2008). Academic buoyancy: towards an understanding of students’ everyday academic resilience. J. Sch. Psychol. 46, 53–83. doi: 10.1016/j.jsp.2007.01.002, 19083351

[ref56] Martin A. J. Marsh H. W. (2020). Investigating the reciprocal relations between academic buoyancy and academic adversity: evidence for the protective role of academic buoyancy in reducing academic adversity over time. Int. J. Behav. Dev. 44, 301–312. doi: 10.1177/0165025419885027

[ref57] Mercer S. (2017). Positive psychology in SLA: an agenda for learner and teacher wellbeing. Aus. Rev. Appl. Lingui. 40, 108–120. doi: 10.1075/aral.40.2.02mer

[ref58] Mercer S. (2021). An agenda for well-being in ELT: an ecological perspective. ELT J. 75, 14–21. doi: 10.1093/elt/ccaa062

[ref59] Mercer S. Gregersen T. (2023). Transformative positive psychology in the acquisition of additional languages. J. Multiling. Multicult. Dev. 46, 2671–2686. doi: 10.1080/01434632.2023.2194869

[ref60] Mercer S. MacIntyre P. D. (2014). Introducing positive psychology to SLA. Stud. Second. Lang. Learn. Teach. 4, 153–172. doi: 10.14746/ssllt.2014.4.2.2

[ref61] Nosratinia M. Saveiy M. Zaker A. (2014). EFL learners’ self-efficacy, metacognitive awareness, and use of language learning strategies: how are they associated? Theor. Pract. Lang. Stud. 4, 1080–1092. doi: 10.4304/tpls.4.5.1080-1092

[ref62] Oladrostam E. Rahmati T. Nushi M. (2025). A third-wave positive psychology approach to language teachers’ self-efficacy, self-concept, and motivation. Lang. Teach. Res. doi: 10.1177/13621688251352270

[ref63] Oxford R. L. (2016). Teaching and researching language learning strategies: self-regulation in context. Abingdon: Routledge.

[ref64] Pan Z. (2019). Struggling between national pride and personal empowerment: the language ideologies held by Chinese university students towards China English. Lingua 227:102702. doi: 10.1016/j.lingua.2019.06.003

[ref65] Podsakoff P. M. MacKenzie S. B. Lee J.-Y. Podsakoff N. P. (2003). Common method biases in behavioral research: a critical review of the literature and recommended remedies. J. Appl. Psychol. 88, 879–903. doi: 10.1037/0021-9010.88.5.879, 14516251

[ref66] Putwain D. W. Daumiller M. Hussain T. Pekrun R. (2024). Revisiting the relation between academic buoyancy and coping: a network analysis. Contemp. Educ. Psychol. 78:102283. doi: 10.1016/j.cedpsych.2024.102283

[ref67] Putwain D. W. Gallard D. Beaumont J. (2020). Academic buoyancy protects achievement against minor academic adversities. Learn. Individ. Differ. 83–84:101936. doi: 10.1016/J.LINDIF.2020.101936

[ref68] Putwain D. W. Wood P. (2023). Riding the bumps in mathematics learning: relations between academic buoyancy, engagement, and achievement. Learn. Instr. 83:101691. doi: 10.1016/j.learninstruc.2022.101691

[ref69] Rost D. H. Feng X. (2024). Academic self-concept wins the race: the prediction of achievements in three major school subjects by five subject-specific self-related variables. Behav. Sci. 14:40. doi: 10.3390/bs14010040, 38247692 PMC10813676

[ref70] Russ H. Sibley L. Flegr S. Kuhn J. Hoogerheide V. Scheiter K. . (2025). Does distributing non-interactive teaching contribute to learning? Students’ academic self-concept and work ethic matter. Learn. Individ. Differ. 120:102687. doi: 10.1016/j.lindif.2025.102687

[ref71] Sang Y. (2023). China’s standards of English language ability: voice from english teachers at Chinese universities. SAGE Open 13:21582440231205434. doi: 10.1177/21582440231205434

[ref72] Sarstedt M. Hair J. F. Cheah J.-H. Becker J.-M. Ringle C. M. (2019). How to specify, estimate, and validate higher-order constructs in PLS-SEM. Australas. Mark. J. 27, 197–211. doi: 10.1016/j.ausmj.2019.05.003

[ref73] Seligman M. E. (2011). Flourish: a visionary new understanding of happiness and well-being. New York, NY: Simon and Schuster.

[ref74] Seligman M. E. (2018). The hope circuit: a psychologist’s journey from helplessness to optimism. London: Hachette UK.

[ref75] Seligman M. E. Csikszentmihalyi M. (2000). Positive psychology. An introduction. Am. Psychol. 55, 5–14. doi: 10.1037//0003-066x.55.1.5 11392865

[ref76] Song Y. (2023). Strategies for improving the passing rate of CET-4 and CET-6. Adult High. Educ 5, 50–55. doi: 10.23977/aduhe.2023.050610

[ref77] Sorjonen K. Ingre M. Melin B. Nilsonne G. (2024). Questioning the reciprocal effects model of academic self-concept and achievement: a reanalysis of a meta-analysis of longitudinal studies and a simulation. SAGE Open 14. doi: 10.1177/21582440241292826

[ref78] Souzandehfar M. Ahmed Abdel-Al Ibrahim K. (2023). Task-supported language instruction in an EFL context: impacts on academic buoyancy, self-esteem, creativity, and language achievement. Asian. J. Second. Foreign. Lang. Educ. 8:43. doi: 10.1186/s40862-023-00218-0

[ref79] Steinberg O. Kulakow S. Raufelder D. (2024). Academic self-concept, achievement, and goal orientations in different learning environments. Eur. J. Psychol. Educ. 39, 3893–3917. doi: 10.1007/s10212-024-00825-6

[ref80] Syafri F. Rafli Z. Emzir E. (2019). The implementation of cognitive academic language learning approach-based Elena: an analysis of introduction to linguistics course in Eleventh conference on applied linguistics (CONAPLIN 2018), Dordrecht: Atlantis press, 186–193.

[ref81] Tuna S. M. (2024). Fixed mindset, achievement goals, reconceptualized L2 motivational self system, academic buoyancy, resilience and success of English preparatory program students at Turkish EMI universities: a structural equation modeling study. Ankara: Bilkent Universitesi.

[ref82] Ulfah K. Supriandi A. Setiawan N. A. (2024). Academic self concept and esteem support in mathematics students: do they affect academic buoyancy. PAEDAGOGY: Jurnal Ilmu Pendidikan dan Psikologi 4, 244–256. doi: 10.51878/paedagogy.v4i3.3321

[ref83] Wang N. (2024). How does basic psychological needs satisfaction contribute to EFL learners’ achievement and positive emotions? The mediating role of L2 self-concept. System 123:103340. doi: 10.1016/j.system.2024.103340

[ref84] Wang Y. Derakhshan A. Zhang L. J. (2021). Researching and practicing positive psychology in second/foreign language learning and teaching: the past, current status and future directions. Front. Psychol. 12:731721. doi: 10.3389/fpsyg.2021.731721, 34489835 PMC8417049

[ref85] Weißenfels M. Hoffmann D. Dörrenbächer-Ulrich L. Perels F. (2022). Linking academic buoyancy and math achievement in secondary school students: does academic self-efficacy play a role? Curr. Psychol., 42, 23422–23436. doi: 10.1007/s12144-022-03488-y, 35874963 PMC9295088

[ref86] Wu Q. (2024). Academic buoyancy, hope, and behavioral engagement in learning English as a foreign language: a mediation model. Int. J. Sci. Res. Arch. 13, 281–292. doi: 10.30574/ijsra.2024.13.1.1629

[ref87] Wu Y. Kang X. (2023). The mediating role of academic self-concept in the linkage between teacher support and academic proficiency among secondary efl learners. Eur. J. Educ. Stud. 10. doi: 10.46827/ejes.v10i8.4922

[ref88] Xiao G. Liu H. (2025). Intrapersonal strengths and interpersonal support: predicting academic buoyancy through psychological capital and growth mindset. Front. Psychol. 16:1584343. doi: 10.3389/fpsyg.2025.1584343, 40746451 PMC12311807

[ref89] Yang D. (2023). Exploring EFL students’ academic buoyancy in Chinese context. Front. Educ. Res. 6:61710. doi: 10.25236/FER.2023.061710

[ref90] Zhai K. (2025). Exploring the relationships between academic buoyancy, engagement, and achievement in English reading among EFL learners. SAGE Open 15. doi: 10.1177/21582440251357156

